# Complicated community-acquired methicillin-resistant *Staphylococcus aureus* pancarditis with cardiac pseudoaneurysm in a healthy child: A case report

**DOI:** 10.1016/j.ijscr.2020.10.085

**Published:** 2020-10-29

**Authors:** Nada A. Aljassim, Nabeel Almashraki, Mohamed Tageldein, Omer Tamimi, Mohamed S. Kabbani, Jihad Zahraa, Mohammed Alshehri

**Affiliations:** a Department of Pediatric Critical Care, Critical Care Center, King Fahad Medical City, P.O. Box. 59046, Riyadh, 11525, Saudi Arabia; b Department of Pediatrics Cardiology, King Salman Cardiac Center, King Fahad Medical City, Riyadh, Saudi Arabia; c Department of Cardiac Sciences, Division of Pediatric Cardiac ICU, MC 1423, King Abdulaziz Medical City, Ministry of National Guard, P.O. Box: 22490, Riyadh, 11426, Saudi Arabia; d Department of Pediatrics Infectious Diseases, King Fahad Medical City, Riyadh, Saudi Arabia

**Keywords:** CA-MRSA, community acquired methicillin-resistant *Staphylococcus aureus*, IE, infective endocarditis, LV, left ventricle, MV, mitral valve, PMVL, posterior mitral valve leaflet, Pancarditis, Pseudoaneurysm, Methicillin-resistant, *Staphylococcus aureus*

## Abstract

**Introduction:**

Infective endocarditis is more prevalent among children with congenital heart diseases as compared to healthy children. Community-acquired methicillin-resistant *Staphylococcus aureus* is a causative pathogen of infective endocarditis, and it rarely causes pancarditis in healthy children. The clinical decision for surgical intervention of left-sided heart vegetation is challenging despite the availability of management guidelines.

**Presentation of case:**

We report a case of a previously healthy 12-year-old girl who presented with aggressive endocarditis secondary to community-acquired methicillin-resistant *Staphylococcus aureus* infection, with left-sided vegetation, mitral valve regurgitation, pancarditis, brain abscess, and stroke. She underwent an emergency vegetectomy and mitral valve repair. Three weeks after the first surgery, she developed left ventricular pseudoaneurysm that required life-saving surgical intervention. The child gradually recovered and was discharged home with acceptable cardiac function and mild neurological deficit.

**Discussion:**

Pancarditis, especially with an aggressive progression resulting in intracardiac pseudoaneurysm, is rarely reported in healthy children. The definition of the optimal timing of surgical intervention in pediatric infective endocarditis management is lacking and the clinical decision-making process remains challenging. The development of left ventricular pseudoaneurysm is serious and also needs an immediate intervention, given the high risk of its rupture and subsequent devastating outcomes.

**Conclusion:**

Community-acquired methicillin-resistant *Staphylococcus aureus* is an etiology for aggressive infective pancarditis in a healthy child, leading to an intracardiac pseudoaneurysm. Emergency surgical interventions should be considered in children with left-sided vegetation to prevent devastating consequences.

## Highlights

• MRSA rarely causes infective pancarditis in healthy children.
• Optimal timing of surgical intervention is unclear in the guidelines.
• A 12-year-old otherwise healthy girl presented with infective pancarditis.
• Despite infection control, she developed a cardiac pseudoanerysm.
• Emergency surgical interventions are crucial in pediatric left-sided pancarditis.

## 1 Introduction

Pediatric infective endocarditis (IE) is a life-threatening infection that usually affects children with pre-existing cardiac conditions. Its incidence in children with a normal heart structure is 8–35.4% [1,2]. The most common pathogens responsible for pediatric IE are *Streptococcus viridans* and *Staphylococcus aureus*. Reports suggest that the incidence of community-acquired methicillin-resistant *S. aureus* (CA-MRSA) is increasing among healthy children [3]. However, reports on pediatric bacterial pancarditis are rare [4,5].

Cardiac complications associated with IE and pancarditis can be intra-cardiac (e.g., pseudoaneurysm) or extra-cardiac (e.g., central nervous system [CNS] embolization resulting in stroke or CNS infection) [2,3,6]. Despite the availability of IE management guidelines and advances in the management of associated complications [2,7], the mortality rate remains high (up to 30%). It is becoming even higher in the presence of neurological complications [8,9]. Furthermore, some of the available pediatric guidelines on IE and pancarditis management do not define the optimal time of surgical interventions clearly. Given the risks associated with surgical interventions, clinical-decisions on the appropriate timing of these interventions remain challenging [10,11].

We report about a 12-year-old girl presenting with complicated CA-MRSA-pancarditis to a tertiary government hospital. Despite controlling the infection, she developed few complications that necessitated an emergent lifesaving surgery (within 24 h) to achieve acceptable cardiac and neurological recovery. The work herein has been reported in line with the SCARE criteria.21 [12].

## 2 Presentation of case

A 12-year-old girl, with no predisposing medical conditions, initially presented to a local health care service with a three-day history of fever, headache, vomiting, and altered mental status. As meningoencephalitis was suspected, she was first treated with intravenous ceftriaxone and acyclovir for seven days then along with vancomycin for three days. While the fever initially subsided for two days, her condition subsequently deteriorated with more lethargy, headache, high-grade fever, vomiting, diarrhea, abdominal pain, and dark yellow urine. Brain computed tomography (CT) scan revealed multiple scattered small brain abscesses. Thus, she was transferred to our hospital, a tertiary governmental hospital, by ambulance for further management. Other medical and surgical histories were unremarkable, and the family members had a fair psychosocial status with no cardiac diseases.

Her vital signs at presentation included a body temperature, blood pressure, heart rate, respiratory rate, and oxygen saturation at room air (SpO_2_) of 39.5 °C, 105/70 mm Hg, 130–150 beats/min, 30 breaths/min, and 95–100%, respectively. Physical examination revealed an ill-appearing, hypoactive child with pallor and small erythematous macules on the palms and soles that were consistent with the Janeway lesions. She had mild respiratory distress and cold extremities associated with muffled heart sounds on chest auscultation. She also had generalized abdominal tenderness and hepatomegaly. Neurological examination was initially unremarkable except for lethargy.

**Fig. 1:**
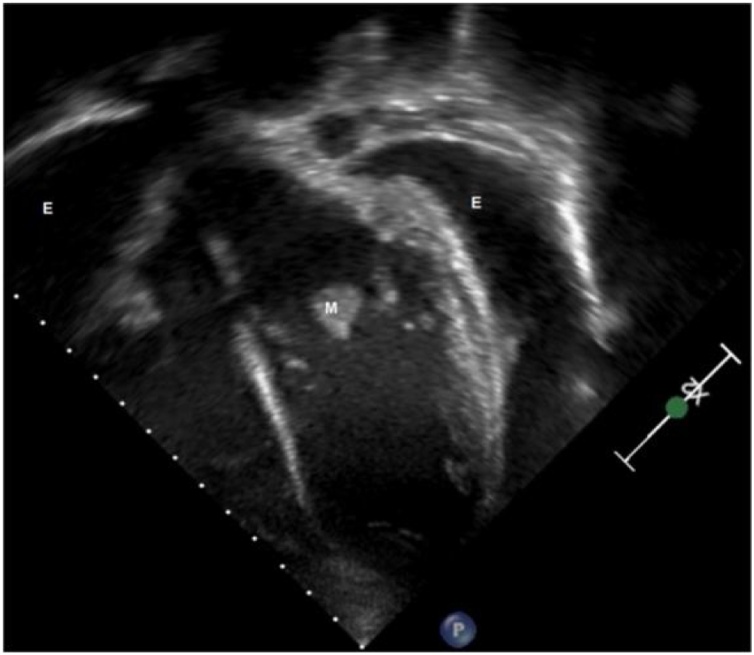
Posterior and anterior pericardial effusion (E), The vegitation attached to mital valve and submital apperatus (M).

**Fig. 2:**
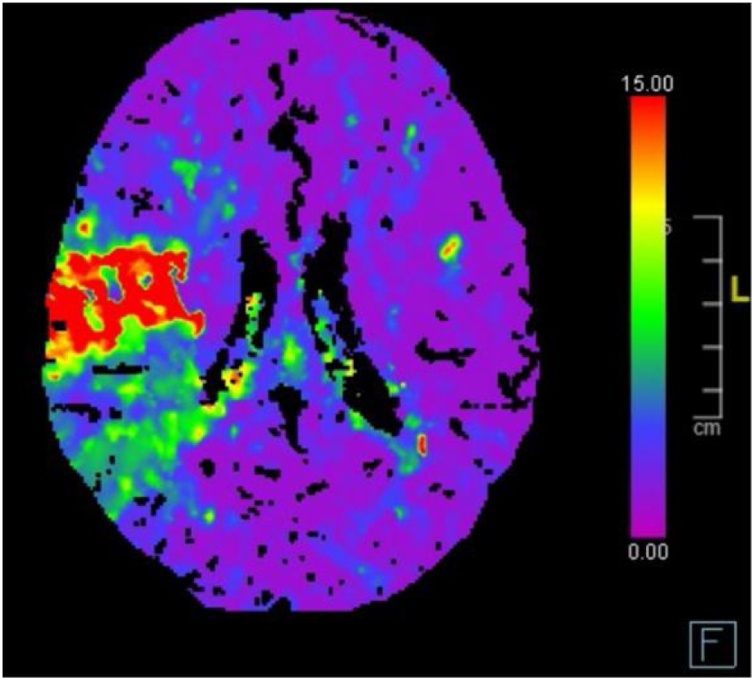
CT scan of brain with perfusion study shows right Middle Cerebral Artery and Anterior Cerebral Artery territorial involvement.

**Fig. 3:**
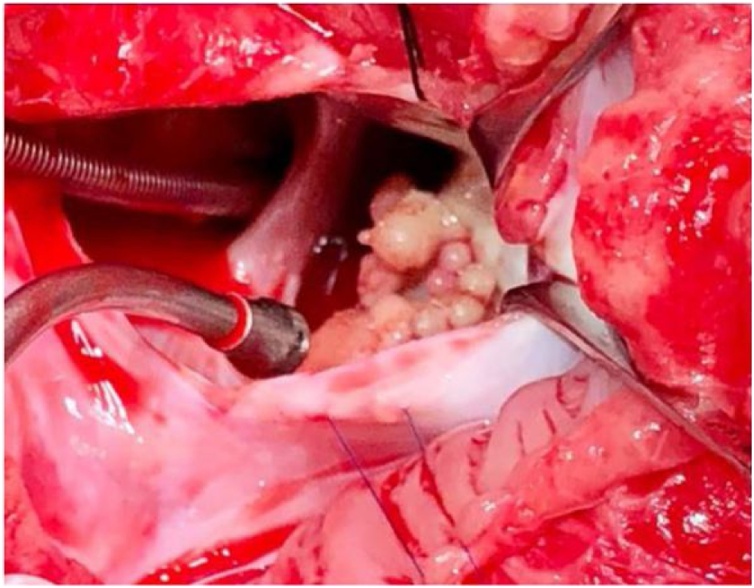
Left ventricle vegitation.

The complete blood count, serum electrolyte levels, hepatic and kidney function, and coagulation profile were normal; however, she had elevated levels of serum inflammatory marker levels. Two consecutive daily peripheral blood cultures yielded MRSA. Laboratory investigations for immunodeficiency and connective tissue diseases were unremarkable. We initiated the patient on intravenous broad-spectrum antimicrobials, including vancomycin (60 mg/kg/day) and gentamicin (5 mg/kg/day). She was stable, but had signs of mild heart failure. Chest radiography revealed significant cardiomegaly with bilateral pleural effusion, while trans-thoracic echocardiography (TTE) revealed two vegetations in the left ventricle (LV): one was attached to the anterior mitral valve (MV) leaflet with significant MV regurgitation and the other was an elongated mass attached to the MV chordae, without any other vegetation in the aortic valve or the right side of the heart (Fig. 1). Serial TTE studies revealed pericardial effusion that increased in size within 48 h, causing right atrial wall collapse. Therefore, a multidisciplinary team meeting was held, with decision of performing pericardiocentesis, and scheduled a cardiovascular surgery for the next day for vegetation removal. After obtaining high-risk consents, an interventional cardiologist performed pericardiocentesis with no complications, and 845 mL of sterile serosanguinous fluid was drained, which contained numerous acute inflammatory cells (mainly neutrophils) without evidence of malignancy or microorganisms. On the same day, the child suddenly developed a slurred speech and left-sided hemiparesis associated with increased distress and increased oxygen requirement. Brain CT confirmed the presence of multiple small brain abscesses with a new right hemisphere infarction consistent with an infarction in the territories of the right middle and anterior cerebral arteries (Fig. 2). She was managed conservatively as multiple teams advised include the pediatric neurology, the neurosurgery, and the stroke team. Due to extensive LV vegetation, progressive congestive heart failure, and life-threatening thromboembolic phenomena, a pediatric cardiovascular surgeon performed an emergency surgical LV vegetectomy with MV repair the next morning. Anesthesia was induced smoothly, and her airway was intubated. After median sternotomy, a 7-mm-thick hyperemic pericardium that adhered to the myocardium and few patchy areas of blood clots within a thick fibrinous inflammatory layer covering the myocardial surface. These findings indicated pancarditis. During standard cardiopulmonary bypass, the heart was opened and the left atrium was accessed through transeptal approach. Multiple vegitectomy were done from the posterior MV leaflet (PMVL) (Fig. 3). Most of the primary chordae of the posterior mitral leaflets where eaten out and ruptured as well as some of the anterior mitral leaflet. Those were repaired using the Matrix Patch (Equine epicardial patch from autotissue, Berlin, Germeny) and GORE-TEX suture (W.L. Gore & Associate, Arizona, USA) artificial chordae implantation. A large abscess cavity lateral to the anterior papillary muscle and extending deep into the myocardium was evacuated and cleaned. This approach was attempted to avoid replacing the valve. TEE after surgerry showed mild mitral regurgitation. Postoperatively, the child was transferred to the pediatric cardiac intensive care under sedation and intubated. She developed vasodilatory shock, and required vasoactive medications for 48 h, then she was weaned off them. Repeated TTE revealed mild mitral regurgitation with no other intracardiac vegetation and a mildly impaired LV systolic function associated with trivial pericardial effusion. Within a week of surgery, she was extubated and discharged from the ICU for IE management in the pediatric ward.

**Fig. 4:**
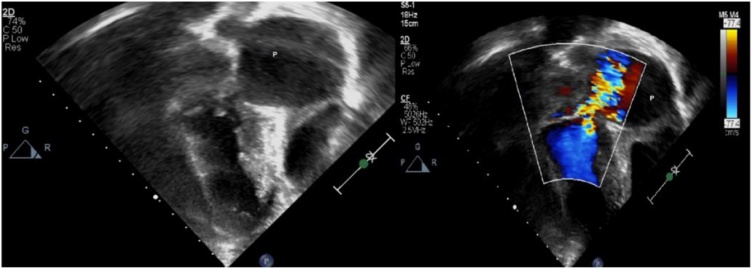
2D and color doppler of the pseudoaneurysm (P) with color aliasing at the connection with left ventricle.

Follow up TTE were performed in the next three weeks which revealed a progressive localized dilatation in the LV at the posterior MV attachment site, consistent with an LV pseudoaneurysm (Fig. 4). In later days, the child suddenly developed pericardial tamponade that needed resuscitation with fluid boluses, vasoactive medication use and intubation. Thus, an emergency intervention was performed for the second time, in which a femo-femoral cardiopulmonary bypass was deployed before opening the sternum. The pseudoaneurysm was contained and repaired by excision, and the GORE-TEX patch closure was buttressed with Teflon sutures. The hemopericardium was evacuated successfuly. She had smooth post operative course and extubated successfuly then transfered to the pediatric cardiology ward. After 4 weeks of IE treatment, the child recovered with proper valvular and cardiac function and residual left-sided hemiparesis, and was discharged on Captopril.

## 3 Discussion

The incidence of MRSA among *S. aureus*-infected children in Riyadh (Saudi Arabia) is 23.2% [13], which is in-line with the incidence that was reported by international reports [14]. CA-MRSA infections are increasing and are associated with invasive infections in immunocompetent children; however, cases of bacterial pancarditis secondary to MRSA are rare [4,5,15]. Our patient was previously healthy. Her presentation was consistent with that of complicated CA-MRSA pancarditis with multiple brain abscesses, LV vegetation, cerebrovascular infarction, and later a ruptured ventricular pseudoaneurysm. She had no evidence of previous primary infectious foci or immunodeficiency. Her management was challenging. Delay in intervention of such cases can lead to devastating clinical consequences or even death [8,9,16]. In our case, pericardiocentesis was performed before cardiac surgery because the child was symptomatic with signs of active infection to give more time for antimicrobial treatment. Unfortunately, she developed a stroke, which is a known result of left-sided heart vegetation and is unlikely to be related to pericardiocentesis. The next morning, an emergency vegetectomy was performed to prevent emboli recurrence. This decision was more difficult due to the high risk of hemorrhagic transformation of the brain infarct.

Various specific indications for surgical intervention are described in different IE management guidelines without clear timing in all of the guidelines [2,7,17]. The 2015 American Heart Association Pediatric IE Guidelines does not specify the most suitable timing for surgery, and leaves it to the multidisciplinary team’s discretion [2]. Conversely, the 2015 European Society of Cardiology (ESC) guidelines for the management of IE classify the timing of surgery into emergency (within 24 h) or urgent (within a few days) depending upon specific criteria, which could make clinical decision a more comfortable and faster process [7]; this is similar to the timing advised in the 2016 American Association for Thoracic Surgery (AATS) guidelines 17]. Kang et al. conducted a randomized trial in 2012 to compare early surgery (within 48 h) and conventional treatment in adults presenting with left-sided IE and extensive vegetation, and reported better outcomes for early surgery in terms of mortality and embolic stroke [18]. Furthermore, a systematic review on IE patients revealed that early surgery was associated with lower in-hospital and long-term mortality rates than non-early surgery [19]. However, no recommendations supports the inclusion of early surgery in the pediatric IE management guidelines, or evidence that prophylactic surgery can prevent emboli [2,7]. Prompt clinical decisions that weigh the risks and benefits of intervention can save the patient's life. Moreover, cardiac pseudoaneurysm has been reported as an early and late complication of IE but rare in children [20]. It can occur in the right ventricle, septum, or LV, (where it carries a higher risk of rapid growth and rupture). Early surgical intervention for LV pseudoaneurysm can result in better outcome, as in our patient. Therefore, a close follow-up of MRSA pancarditis cases using TTE or cardiac magnetic resonance imaging for early detection of post-operative complications is recommended.

## 4 Conclusion

We recommend immediate surgical intervention in children with pancarditis that involves left-sided vegetation, once indicated, and a close follow-up using serial echocardiography to detect complications early. Early surgical intervention of LV pseudoaneurysm is strongly favored, given the risk of a devastating outcome if lifted without intervention.

## Conflicts of interest

No conflict of interest.

## Funding

No founding or grants provided for this case report publication.

## Ethical approval

Not applicable.

## Consent

Written informed consent was obtained from the patient for publication of this case report and accompanying images. A copy of the written consent is available for review by the Editor-in-Chief of this journal on request.

## Author contribution

Nada Aljassim: First author and corresponding author. Responsible for initiation of reporting, writing editing the manuscript and literature review.

Nabil Almashraki: Patient course and data collection, literature review.

Mohamed Tageldein: cardiac surgeon contributed in operation details and patients course, literature & manuscript review.

Omer Tamimi: pediatric cardiologist shared patient’ s echo images,literature & manuscript review.

Mohamed S. Kabbani: literature & manuscript review.

Jihad Zahraa: literature & manuscript review.

Mohammed Alshehri: review of infectious disease literature & manuscript review.

## Registration of research studies

Not applicable.

## Guarantor

I am fully responsible for the work and have access to the data, and controlled the decision to publish.

Dr. Nada Aljassim

Corresponding author

## Provenance and peer review

Not commissioned, externally peer-reviewed.
